# Exploring Factors Affecting Impostor Syndrome among Undergraduate Clinical Medical Students at Chiang Mai University, Thailand: A Cross-Sectional Study

**DOI:** 10.3390/bs13120976

**Published:** 2023-11-27

**Authors:** Purichaya Shinawatra, Chayada Kasirawat, Phichittra Khunanon, Sorrathorn Boonchan, Siripit Sangla, Benchalak Maneeton, Narong Maneeton, Suttipong Kawilapat

**Affiliations:** 1 Faculty of Medicine, Chiang Mai University, Chiang Mai 50200, Thailand; purichaya_s@cmu.ac.th (P.S.); chayada_kas@cmu.ac.th (C.K.); phichittra_kh@cmu.ac.th (P.K.); sorrathorn_boon@cmu.ac.th (S.B.); siripit_s@cmu.ac.th (S.S.); 2 Department of Psychiatry, Faculty of Medicine, Chiang Mai University, Chiang Mai 50200, Thailand; narong.m@cmu.ac.th (N.M.); suttipong.kawilapat@cmu.ac.th (S.K.)

**Keywords:** impostor syndrome, impostor phenomenon, medical students, depression, anxiety

## Abstract

Impostor syndrome is a psychological condition that inhibits individuals’ ability to recognize their achievements such that they fear being exposed as forgers. It is common in medical students, particularly in the early stages of clerkship training while transitioning from preclinical to clinical training. This cross-sectional study assessed the prevalence and associated factors of the imposter phenomenon among medical clinical students using the Clance Impostor Phenomenon Scale (CIPS), focusing on sociodemographic characteristics, mental health status, and occurrence of the impostor phenomenon. Out of 228 undergraduate clinical-year medical students, 108 (47.4%) reported experiencing the impostor phenomenon. The results from the multivariable analysis showed that high levels of stress (adjusted odds ratio = 2.315; 95% confidence interval = 1.105–4.853), anxiety (6.462; 1.374–30.392), and depression (4.219; 1.448–12.290) were significantly associated with an increased risk of experiencing the impostor phenomenon. We found no difference between participants in the early or later years of clerkship training. The study highlights the prevalence of impostor syndrome among medical students and its link to mental health issues. Addressing this issue through education, mentorship, systemic problem solving, normalizing failure, and monitoring and treating mental health issues could help students reach and realize their full educational and professional potential.

## 1. Introduction

The impostor syndrome, often called the impostor phenomenon, impostor experience, impostorism, or fraud syndrome, is a psychological state in which people doubt their ability to complete a task even when they have evidence to the contrary. Impostor syndrome can cause anxiety, despair, and low self-esteem in those who feel inauthentic and continually worry that they are inadequate [1,2,3,4,5]. Even though imposter syndrome is not a recognized mental health disorder, it severely affects people’s well-being. Impostor syndrome can cause high achievers to mistrust their skills, fail to acknowledge their accomplishments, and worry about being exposed as fraudsters. Clance and Imes first theorized the phenomenon in 1978, which came to the public’s attention in 1985 when Clance’s book was published [6,7]. Initially, it was believed to be shared among highly successful professional women, but current research has shown that it may happen to a variety of people, especially those who work in the medical area, such as medical students, resident physicians, and physicians [1,2,3,4,5]. Due to their frequent exposure to high achievers and their work environment’s intense competition, these people may experience the impostor phenomenon more intensely, which could hurt their ability to perform in their professional roles. The impostor phenomenon received further attention after Mike Cannon-Brookes, the billionaire entrepreneur and co-founder of the software business Atlassian, spoke in a TED talk about it in 2017. The term “Impostor Phenomenon” grew in popularity, and talks about it now cover psychology and other fields [8].

Numerous reasons contribute to impostor syndrome’s prevalence, and its risk factors may exist even before one enrolls as a medical student. According to earlier research, medical students, trained physicians, and physicians frequently experience the imposter phenomenon, with incidence rates ranging from 22% to 70.3% [1,4,5,9,10,11,12,13,14]. The preclinical to clinical phases are when this tendency is more prevalent among people transitioning into new careers. The development of imposter sentiments may be influenced by several variables, including gender [4,5,6,9,15,16], the training-phase shift from preclinical to clinical, the difficulty of clinical learning, and competency-based curricula [11,12,14,17]. Additionally, research has linked the impostor phenomenon to mental illnesses like perception of social expectation in the role of a medical student, poor coping strategies, low self-esteem, maladaptive perfectionism, poor sleep quality, anxiety, depression, burnout, quitting training, and suicidal ideation [1,3,11,12,13,14,18]. Therefore, sociodemographic characteristics and mental health are two important factors associated with the impostor phenomenon. Despite some indications that the imposter phenomenon is common in our culture, research on the causes is lacking. Consequently, this knowledge gap offers a fascinating chance for research.

We conducted a thorough literature review that included studies from various regions, focusing on the prevalence, causes, classification, and psychosocial determinants of this phenomenon, as described in previous publications [1,4,5,9,10,17], to investigate the phenomenon of impostor syndrome in our nation. We also wanted to investigate how social and cultural aspects of our nation may affect how impostor syndrome manifests itself and its effects. To accomplish this, we conducted a survey- or interview-based examination of participants’ experiences and perceptions of impostor syndrome to spot any notable trends or variances in our sample. This research endeavored to explore the prevalence and influencing factors of imposter syndrome among medical students who were in their clinical year of training at Chiang Mai University, Thailand. Hypothesizing that sociodemographic characteristics and mental health conditions play a role in the frequency of imposter syndrome among undergraduate medical students in their clinical year, our objective was to investigate this phenomenon. Specifically, we aimed to analyze factors such as age, gender, socioeconomic status, and family background, while also assessing the correlations between mental health conditions and imposter syndrome. Employing a cross-sectional study design, we planned to conduct an online survey using a Thai version of a validated imposter syndrome scale to gather comprehensive data at a specific point in time. Through this methodology, we sought to contribute valuable insights into the prevalence and associated factors of imposter syndrome in this student population.

## 2. Materials and Methods

### 2.1. Study Design and Study Participants

This study was an observational cross-sectional study conducted among undergraduate clinical medical students during clerkship training between November 2021 and January 2022. Our nation’s first three years of the six-year Doctor of Medicine curriculum are devoted to systems-based undergraduate preclinical training. Medical students undertake clerkships during the latter three years of the six-year medical education program. The program’s focus for medical students is the fourth, fifth, and sixth years of medical school—the clinical training and clinical rotations or clerkships. Medical students practice in major and minor rotations and participate in a clerkship during this period. Major rotation is a term used to describe training in larger or more complicated wards, such as internal medicine, surgery, obstetrics and gynecology, and pediatrics. These wards demand intense concentration and hard work and are fast-paced environments. Minor rotation, on the other hand, entails alternating between smaller or less complex wards in addition to the major wards already described. These wards include those for ophthalmology; ear, nose, and throat (ENT); family medicine; psychiatry; orthopedics; and rehabilitation.

### 2.2. Data Collection Process

De-identified online surveys were conducted to collect data using the Research Electronic Data Capture (REDCap) platform. This study gathered information on sociodemographic traits, mental health issues, and other pertinent details. Throughout the recruiting period, from November 2021 to January 2022, each medical class representative was tasked with providing online links and QR codes to invite participants to complete the questionnaire. Prior to responding to the questionnaire, the participants were shown a pamphlet detailing the study’s objectives and asked for their consent. Participation was therefore optional and anonymous, and responses were treated with the utmost confidentiality. After completing the surveys, the participants were urged to get in touch with a psychiatrist for medical care if they scored highly for stress, anxiety, or suicidal ideation.

### 2.3. Ethical Considerations and Confidentiality

The study protocol received ethical approval from the Research Ethics Committee of the Faculty of Medicine, Chiang Mai University (REC no. 267/2021). A consent form and an information sheet were given to the participants. The authors guaranteed the privacy of any information gathered and adhered to the Declaration of Helsinki’s guiding principles.

### 2.4. Assessment Tools

Three separate segments of an online questionnaire were used in the current investigation. The participants’ sociodemographic data were covered in the first segment, and the prevalence of the impostor phenomenon was assessed in the second section using the Clance Impostor Phenomenon Scale [19]. The validated psychometric tools (Thai versions), including the Thai Perceived Stress Scale 10 for measuring perceived stress [20,21], the Generalized Anxiety Disorder scale for measuring perceived anxiety [22,23], and the Patient Health Questionnaire for measuring depression [24,25], were all applied in the final portion, which was devoted to psychological evaluations.

#### 2.4.1. Sociodemographic Questionnaire

Sociodemographic data which previously suggested potential associations with the imposter phenomenon were collected in this study, including grade point average (GPA) [26,27], gender [4,5,6,9,15,16], medical year [17], ward rotation [28,29,30,31], birth order [32,33], and number of siblings [34].

#### 2.4.2. The Clance Impostor Phenomenon Scale (CIPS)

The Clance Impostor Phenomenon Scale, a 20-item questionnaire, was used for screening of the imposter phenomenon. The participants responded to each item on a Likert scale from 1 (not at all true) to 5 (extremely true). The CIPS score ranges from 20 to 100 points, a cut-off point of 60 points indicating a high level of impostor experience [35].

To our knowledge, this instrument had not previously been translated into a Thai version. Therefore, we contacted Professor Pauline Rose Clance and asked permission to use the Clance Impostor Phenomenon Scale in this study. The first team of authors translated the scale into the Thai language, then the second team back-translated from the Thai into English, and the accuracy of the translation was determined through comparison. We then conducted a pilot test of the translation with a small sample of participants after accurately and sensitively translating the scale to avoid translation errors or cultural misunderstandings to make sure that the translation was accurate, understandable, and appropriate for the target audience.

According to Cronbach’s alpha coefficients, the internal consistency of the Thai version was excellent, with an overall scaling coefficient of 0.9153 (item coefficients ranging from 0.9060 to 0.9210). The reliability of individual items ranged from 0.4082 to 0.8701, according to intraclass correlation coefficients (ICCs). Seven items—2, 6, 12, 13, 17, 18, and 20—demonstrated high stable reliability, whereas eight items—3, 5, 7, 10, 11, 14, 15, and 16—showed moderate reliability and five items—1, 4, 8, 9, and 19—showed low reliability. The CFA results demonstrated moderate construct validity, with fair model fit indices (Root Mean Square Error of Approximation, RMSEA = 0.096) or nearly good fit values (comparative fit index, CFI = 0.829; Tucker–Lewis Index, TLI = 0.809) (Table S1).

#### 2.4.3. The Thai Perceived Stress Scale 10 (T-PSS-10)

This study measured stress levels using the Thai Perceived Stress Scale 10 (T-PSS-10) [20], a 10-item version of a self-report questionnaire to assess a person’s thoughts and feelings regarding stressful circumstances they encountered in the preceding month. A 5-point Likert scale ranging from 0 (never) to 4 (very often) was used to rate each item. The overall score varied from 0 to 40 points, with 0 to 13 signifying low stress, 14 to 26 signifying moderate stress, and 27 to 40 signifying high stress. The diagnosis of moderate to high levels of perceived stress was made using a cut-off point of 14 or higher. This Thai version was translated from the original English version [21,36]. The T-PSS-10’s overall Cronbach’s alpha was 0.85. This suggests that the items in the T-PSS-10 measure the same underlying construct of perceived stress, indicating that the scale has strong internal consistency [20].

#### 2.4.4. The Generalized Anxiety Disorder Scale (GAD-7)

The Generalized Anxiety Disorder 7-item scale (GAD-7) was initially developed in English and showed good psychometric properties with a Cronbach’s alpha of 0.92 [22]. In a study with Thai subjects, the GAD-7 Thai version showed good reliability (Cronbach’s alpha coefficient of 0.89) [23]. With this modification, anxiety symptoms in Thai individuals are assessed using the GAD-7 in a way that is both applicable and culturally appropriate. The scale, which measures the frequency and severity of anxiety symptoms during the previous two weeks, consists of seven items. Participants who had high scores on the GAD-7 exhibited severe anxiety. Participants react to each item on a four-point Likert scale ranging from 0 to 3. Scores of 5 to 9 indicate mild anxiety, 10 to 14 indicate moderate anxiety, and 15 to 21 indicate severe anxiety. The total score ranges from 0 to 21 points. The diagnosis of moderate to severe anxiety was made using a cut-off point of 10 or higher.

#### 2.4.5. The Patient Health Questionnaire 9 (PHQ-9)

The PHQ-9 is a self-administered questionnaire frequently used in clinical settings to screen, diagnose, and track depressive episodes in adults. A major depressive episode is defined by the Diagnostic and Statistical Manual of Mental Disorders (DSM-5) criteria, represented by nine questionnaire items. Each item on the PHQ-9 is scored on a scale from 0–3, with “0” denoting no symptoms at all to “3” denoting symptoms nearly every day over the previous two weeks. The overall score is between 0 and 27. None (0–4), mild (5–9), moderate (10–14), moderately severe (15–19), and severe (20–27) are the different levels of depression severity. A score of 10 or higher is the threshold for diagnosing moderate to severe depression [37]. The original PHQ-9 questionnaire is in English, and a translated version in Thai is also available. The Thai version of the PHQ-9 had satisfactory internal consistency (Cronbach’s alpha = 0.79) [24].

### 2.5. Sample Size Calculation

According to a previous investigation, the impostor problem among medical students has a prevalence of about 45.7%. Under the assumptions of a 95% confidence interval (CI) and a 5% error rate, the sample size was calculated using the following equation:

(1) $$n=\frac{\left({Z_{\alpha /2})}^{2}P(1-P\right)}{d^{2}}=\frac{{\left(1.96\right)}^{2}(0.457)(1-0.457)}{{\left(0.05\right)}^{2}}=381.32\;\approx 382,$$

Three hundred and eighty-two medical students at our University Hospital enrolled in the fourth through sixth years of their medical studies was the estimated sample size required for the current study.

We also considered the sample size for internal consistency and intraclass correlation estimation according to the sample size determination method introduced by Bonett [38,39]. The minimal sample size required for the internal consistency estimation was 38 individuals given the assumptions of the 20-item scale with an 80% expected Cronbach’s alpha coefficient, 95% CI, and 10% precision. With the assumptions of 75% expected test–retest reliability, 95% CI, and 20% precision, the sample size for intraclass correlation estimation was calculated as 20 individuals.

### 2.6. Statistical Analysis

Sociodemographic data were presented using descriptive statistics. Frequencies and percentages were reported for categorical variables. The continuous variables were reported as means and standard deviations (SDs) or as medians and interquartile ranges (IQRs) according to the distribution of the data.

To evaluate the internal consistency reliability, stability reliability, and construct validity of the Clance Impostor Phenomenon Scale (CIPS), we used Cronbach’s alpha coefficient analysis [40], test–retest reliability with intraclass correlation coefficients (ICCs) [41], and confirmatory factor analysis (CFA), respectively. Cronbach’s alpha coefficients of 0.70–0.79, 0.80–0.89, and 0.90 or more were regarded as fair, good, or excellent degrees for internal consistency, respectively. ICC values of less than 0.50, 0.50–0.75, 0.75–0.90, and greater than 0.90, were considered poor, moderate, good, and excellent reliability levels, respectively. According to the CFA, a Root Mean Square Error of Approximation (RMSEA) [42] of less than 0.06, a Tucker–Lewis Index (TLI) of 0.95 or more, and a Comparative Fit Index (CFI) of 0.95 or more were all found to be the adequate fit indices of construct validity.

A binary logistic regression analysis was used to examine the association between the imposter phenomenon and potential associated variables. All significant variables in the univariable analysis (*p*-value 0.05) were included in the multivariable analysis with backward elimination. All analyses were performed using Jamovi version 2.2.5 and Stata version 17.

## 3. Results

### 3.1. Student Demographics 

**Figure 1:**
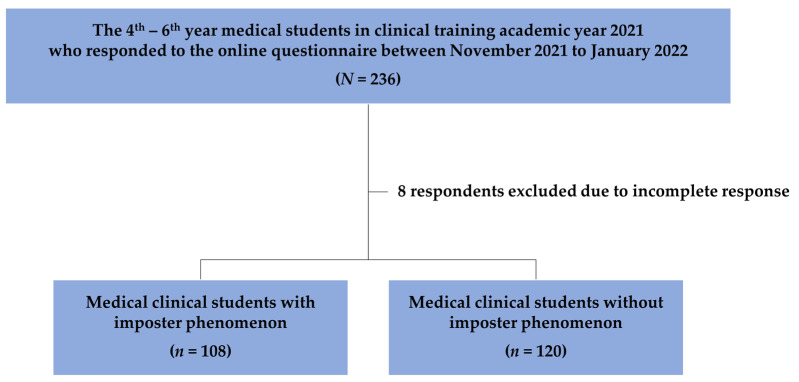
The flow of the study population.

All the study participants were recruited from among the medical students who received training at the University Hospital and two affiliated hospitals in 2021 using a sequential sampling approach. The study did not include any incomplete surveys. Out of the anticipated 382 respondents, 228 medical students responded to the online survey (response rate of 59.7%). Of these, 108 students experienced the imposter phenomenon (47.4%) (Figure 1).

Table 1 provides descriptive statistics for the participants’ sociodemographic data. Between the ages of 21 and 25, women made up the majority of responses (59.6%), while the majority of participants (66.7%) were in their fourth year of undergraduate studies. More than half of the students (59.2%) rotated to major wards like surgery, internal medicine, pediatrics, obstetrics, and gynecology and had a grade point average (GPA) of at least 3.00 (72.8%). Most participants (76.8%) rotated to the University hospital. The majority of participants (62.3%) were firstborns in their families and had siblings (76.8%).

### 3.2. Prevalence and Associated Factors of the Impostor Phenomenon

**Figure 2:**
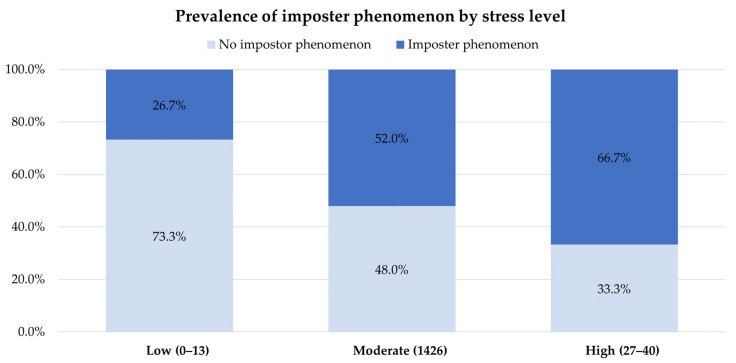
Prevalence of the imposter phenomenon by stress level.

**Figure 3:**
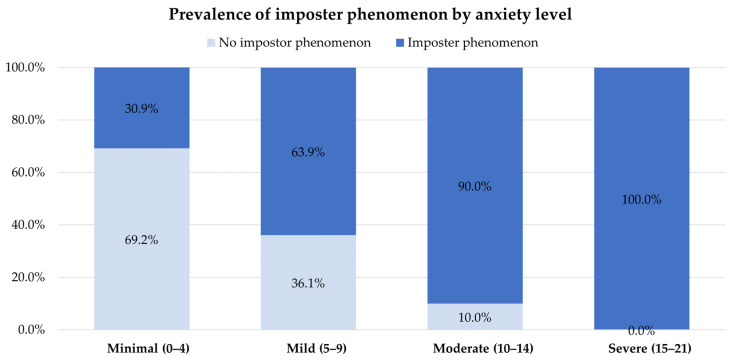
Prevalence of the imposter phenomenon by anxiety level.

**Figure 4:**
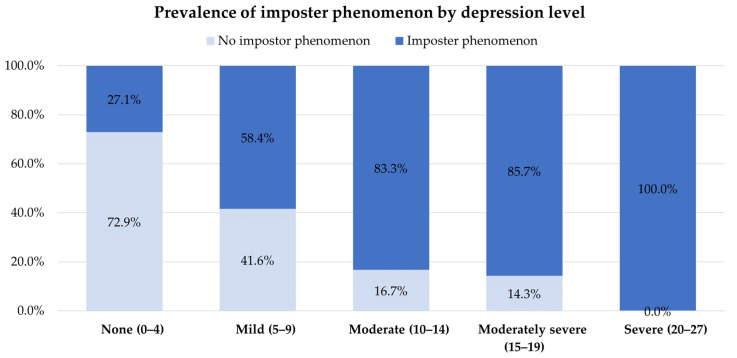
Prevalence of the imposter phenomenon by depression level.

Based on a CIPS score of 61 or higher, 47.4% (108/228) of the individuals were identified as experiencing the imposter phenomenon. We did not find any appreciable differences regarding gender, medical year, ward rotation, location of training, and GPA between the groups with and without the imposter phenomenon. Similarly, there were no notable variations between the groups regarding family background, including sibling status and birth order. However, we discovered correlations of stress, anxiety, and depression with the imposter phenomenon. The clinical medical students with higher scores on stress, anxiety, or depression scales tended to have higher scores on the imposter phenomenon scale (Figure 2, Figure 3 and Figure 4).

According to the univariable analysis, medical students who struggled with stress (odds ratio (OR) = 3.034; 95% confidence interval (CI) = 1.475–6.244), anxiety (OR = 14.241; 95% CI = 3.254–62.323), or depression (OR = 7.667; 95% CI = 2.833–20.751) were more likely to encounter the imposter phenomenon. According to multivariable analysis with backward elimination, all significant variables with a *p*-value less than 0.05 in univariable analysis, including stress, anxiety, and depression, remained as factors significantly related to the imposter phenomenon. Additionally, medical students who had high perceived stress scores (adjusted odds ratio (aOR) = 2.315; 95% CI = 1.105–4.853), anxiety symptoms (aOR = 6.462; 95% CI = 1.374–30.392), and depressive symptoms (aOR = 4.219; 95% CI = 1.448–12.290) were more likely to experience the imposter phenomenon than those who did not (Table 2).

## 4. Discussion

This cross-sectional study designed to explore the imposter phenomenon in clinical medical students was conducted among 228 fourth- to sixth-year medical students. Nearly half of the study’s participants (47.4% (108/228)) had encountered the imposter phenomenon. Individuals who admitted to having impostor sentiments accepted that they had them frequently (92.6% (100/108)), while 7.4% (8/108) experienced them severely. These results imply that the imposter phenomenon may be more common among medical students of our nation and that more research is required among a larger population, particularly in the medical sector. The prevalence of the imposter phenomenon in our study was in the ranges previously reported among medical students around the world [1,3,43,44]. The variation in the imposter phenomenon might be attributable to variations in study environments, cultures, or curricula. 

Several sociodemographic factors previously revealed to be linked with the imposter syndrome include gender, medical year, ward rotation, birth order [32], number of siblings [34], training location, and GPA [26,27]. Previous studies [4,5,6,9,15] demonstrated that women are more susceptible to the imposter syndrome because of institutionalized hurdles and discrimination in their occupations, which causes uncertainty about their skills and a sense of exclusion. These phenomena may also be influenced by societal preconceptions and expectations, since women may feel compelled to uphold preconceived notions of femininity and suffer unfavorable judgment when they deviate from these ideals. However, our study did not find any significant difference in the prevalence of imposter syndrome between genders, which could be related to cultural differences. Regarding women’s rights and freedoms in the region, our nation is renowned for being a relatively liberal and open nation. Many women enjoy a high level of autonomy; access to education, healthcare, and work opportunities; and a generally accessible society that is free from rigid gender roles and expectations. This freedom allows women to express themselves and follow their interests and aspirations freely.

Due to the notion of having insufficient skills and knowledge, as well as the fact that they have not yet fully acclimated to their professional environment or found a sense of belonging within the medical community, impostor syndrome may be more common among medical students during the transition from the preclinical to clinical phases of their training [17] or in their early years of training. Typically, these emotions subside as students acquire experience and become more comfortable in their positions. According to our study, the prevalence of impostor syndrome did not significantly differ between the early and later years of medical school. This might be a result of the medical faculty’s historical culture, effective mentoring programs, and educational framework that prioritizes seniority over juniors to help them succeed in their studies.

Medical students may have tremendous difficulties during clinical rotations, particularly major rotations like internal medicine, surgery, obstetrics, gynecology, or pediatrics, which can exacerbate thoughts of impostor syndrome. These rotations challenge students to take on new tasks and duties if they lack expertise or understanding, function in high-pressure circumstances, and learn new skills quickly. Working with new healthcare teams can make one feel inadequate and self-conscious. However, according to our investigation, there were no appreciable differences between major and minor ward rotations in the prevalence of imposter syndrome. This outcome may be credited to the clear objectives, customized learning activities, and ongoing student and course assessments intended to support instructional changes. Additionally, senior staff, ward staff, and academic teams are crucial in assisting medical students during clinical rotations.

According to previous reports in the literature, primogeniture may come with a sense of obligation to perform well and take on more important duties, perhaps causing the establishment of imposter syndrome and the related emotions of inadequacy and self-doubt. Firstborn children may also experience more scrutiny and comparisons with their siblings, which increases the likelihood of impostor syndrome [32,33]. Nevertheless, there were no appreciable differences in the prevalence of imposter syndrome between different birth orders shown in our study. This finding aligns with a larger body of the literature that contends that the influence of birth order is frequently insignificant or may be fictitious.

The presence of siblings may influence the emergence of imposter syndrome [34]. Constant comparisons between siblings can exacerbate feelings of inadequacy, particularly if one considers the other to be more skilled, accomplished, or intelligent. The sibling who feels inadequate may develop imposter syndrome. On the other hand, healthy sibling relationships can offer emotional support, helpful criticism, and a sense of inclusion, boosting confidence. This was inconsistent with our study, which found no appreciable variations in the prevalence of imposter syndrome between those who had siblings and those who did not. However, our study did not specifically explore the potential impact of positive or negative sibling relationships on the appearance of imposter syndrome.

The medical school environment is often competitive [44], as students’ abilities, performance, and academic competence are constantly assessed. This may lead to anxiety among medical students, self-doubt, and the feeling of being an outsider, that is, to impostor syndrome. Large medical schools may have more students and a more competitive environment, which can pressure students to perform well and lead to feelings of inadequacy and self-doubt. Furthermore, busy faculty members at prestigious medical schools might not have the time to provide each student the individual attention they deserve, which could make students feel isolated. Finally, the name and status of a top medical school may breed expectations of excellence, which, if a student believes they are falling short of these goals, can worsen imposter syndrome. According to our study, the prevalence of impostor syndrome among medical students enrolled at primary or affiliated medical schools was not significantly different.

The imposter syndrome causes students to feel undeserving of their academic accomplishments or good grades and to question their intelligence in contrast to their peers, which can harm measures of a student’s academic performance, like GPA [26]. Even though the previous study showed a correlation between higher levels of the impostor phenomenon and higher GPA, our study found no differences in the prevalence of impostor syndrome among students with various GPA levels, and this conclusion is consistent with another study’s finding that there is no connection between impostor syndrome and GPA [27].

Although some stress is acknowledged as a natural component of medical school and can inspire some people, not all students find stress bearable. Stress is a normal response to challenging situations; however, a previous study found a correlation between chronic stress and anxiety–depressive disorders [10]. The lengthy and difficult nature of medical education is well documented to cause psychological problems in medical students. The intense workload, long study hours, pressure to perform well, and exposure to emotionally taxing events can result in chronic stress. Moreover, chronic stress perhaps raises the chance of developing anxiety or depression [6,8,15,16,17]. Our research revealed a strong link between stress, anxiety, or depression and the imposter syndrome, which interact in a two-way cause-and-effect relationship that can make it difficult to break the cycle in several ways. For instance, impostor syndrome can drive individuals to set impossibly high standards for themselves—a trait called perfectionism. They feel the need to consistently prove their competence, which leads to chronic stress, anxiety, or depression, as they constantly strive to meet these unrealistic expectations. According to the findings of our study, medical students who have psychological problems are associated with imposter syndrome. Impostor syndrome can be exacerbated by stress from medical studies and for other reasons, which can increase feelings of inadequacy and self-doubt in people. People’s cognitive resources may be exhausted under stress, making it more likely that they focus on their perceived flaws rather than their accomplishments. This can start a loop of negative self-talk and strengthen the notion that one’s achievement is not attributable to one’s own abilities and skills but rather to luck or other outside forces. High levels of psychological distress can also lead to a more critical view of oneself and a greater dread of failing. As medical students start to interpret their psychology-related struggles or errors as proof that they are not actually competent, these emotions can exacerbate the imposter syndrome experience.

Based on the study’s findings, we can conclude that strong evidence links mental health issues to the imposter phenomenon. However, the prevalence of the imposter phenomenon among medical students did not support our theory that it occurs more frequently in the early years of clinical training. Furthermore, contrary to our assumption regarding demographic parameters, no significant association between impostor phenomena and general demographic data was discovered.

There were some limitations to this study. First, the response rate of the target population was lower than anticipated (59.7%). We suspected that their workload may have contributed to this issue. Since the sample size of this study did not meet the calculated requirements, it might have provided insufficient statistical power in prevalence estimation. A greater sample size could enhance precision and alter connections. The epidemic made it challenging to track participation in the online survey. In further studies, we suggest using a control group, such as a group with high levels of anxiety and depression but in a less stressful environment, medical students from other nations, or students of other professions (e.g., psychology students, nursing students, or students in the science and technology fields). Finally, selection bias was introduced by using the consecutive sampling approach rather than stratified random sampling. A stratified random sampling approach might lessen bias and produce more reliable connections between variables. 

This study’s main strength was its capacity to examine psychological issues underreported in the literature, such as the imposter phenomenon and others, among medical students. The results of our study can be used as a reference when formulating plans to improve mental health and lessen psychological suffering among medical students. Future studies can investigate the most effective strategies for assisting medical students in avoiding and conquering the imposter phenomenon. The translated version of the CIPS might be utilized to accurately measure the imposter phenomenon in various nation groups because our study used a standardized assessment with strong reliability and reasonable validity. However, since some items of the translated version of the CIPS yielded low reliability, the revision of statements or translation of these items should be conducted to increase its reliability in further uses.

## 5. Conclusions

The impostor phenomenon is common, according to our study on medical students, with over half of the participants reporting having dealt with it. Our study found no statistically significant relationship between GPA, medical year, ward rotation, siblings, birth order, training place, or gender and the impostor phenomenon. The impostor phenomenon and psychological factors, such as high stress, anxiety, and depression, did, however, appear to be closely related. With imposter syndrome, medical students commonly suffer high levels of self-doubt and dread of failing. High stress levels can exacerbate anxiety and depression. This psychological condition can lead to burnout and even suicidal thoughts because of the pressure to achieve and the dread of being discovered as a fraud.

Early preventative screening should be carried out to assist students when they begin their clinical training. To effectively tackle the impostor problem among medical students, it is crucial to prioritize their mental health. Educating students about the impact of the impostor phenomenon on mental health is essential. Additionally, implementing policies and measures that promote good mental health and create a positive learning environment will significantly improve the well-being of medical students. Medical schools should take proactive steps to lower stress levels and foster a healthy learning environment, thereby better preparing students for the challenges of clinical training.

Furthermore, we recommend conducting longitudinal research involving various healthcare professionals and trainees to gain a better understanding of the origins and effects of imposter syndrome in the healthcare industry. This research can provide valuable insights for developing targeted interventions and support systems to address the issue effectively.

## Figures and Tables

**Table 1:** Characteristics of participants and prevalence of impostor phenomenon.

Characteristics	Total (*N* = 228)	Impostor Phenomenon	
		Yes (*n* = 108)	No (*n* = 120)
Gender			
Male	92 (40.4%)	43 (46.7%)	49 (53.3%)
Female	136 (59.6%)	65 (47.8%)	71 (52.2%)
Year of training			
4th year	152 (66.7%)	68 (44.7%)	84 (55.3%)
5th year	54 (23.7%)	28 (51.9%)	26 (48.1%)
6th year	22 (9.6%)	12 (54.5%)	10 (45.5%)
Ward rotations			
Major ward rotation ^a^	135 (59.2%)	67 (49.6%)	68 (50.4%)
Minor ward rotation ^b^	93 (40.8%)	41 (44.1%)	52 (55.9%)
Birth order			
First-born child	142 (62.3%)	69 (48.6%)	73 (51.4%)
Non-first-born child	86 (37.7%)	39 (45.3%)	47 (54.7%)
Participants with siblings			
Yes	175 (76.8%)	82 (46.9%)	93 (53.1%)
No	53 (23.2%)	26 (49.1%)	27 (50.9%)
Place of training			
Medical school	175 (76.8%)	88 (50.3%)	87 (49.7%)
Affiliated medical school 1	22 (9.6%)	7 (31.8%)	15 (68.2%)
Affiliated medical school 2	31 (13.6%)	13 (41.9%)	18 (58.1%)
GPA			
2.00–2.49	11 (4.8%)	4 (36.4%)	7 (63.6%)
2.50–2.99	51 (22.4%)	31 (60.8%)	20 (39.2%)
3.00–3.49	91 (39.9%)	38 (41.8%)	53 (58.2%)
3.50–4.00	75 (32.9%)	35 (46.7%)	40 (53.3%)
Depression level (PHQ-9 score)			
None (0–4)	107 (46.9%)	29 (27.1%)	78 (72.9%)
Mild (5–9)	89 (39.0%)	52 (58.4%)	37 (41.6%)
Moderate (10–14)	24 (10.5%)	20 (83.3%)	4 (16.7%)
Moderately severe (15–19)	7 (3.1%)	6 (85.7%)	1 (14.3%)
Severe (20–27)	1 (0.4%)	1 (100.0%)	0 (0.0%)
Anxiety level (GAD-7 score)			
Minimal (0–4)	133 (58.3%)	41 (30.8%)	92 (69.2%)
Mild (5–9)	72 (31.6%)	46 (63.9%)	26 (36.1%)
Moderate (10–14)	20 (8.8%)	18 (90.0%)	2 (10.0%)
Severe (15–21)	3 (1.3%)	3 (100.0%)	0 (0.0%)
Stress level (PSS-10 score)			
Low (0–13)	45 (19.7%)	12 (26.7%)	33 (73.3%)
Moderate (14–26)	177 (77.6%)	92 (52.0%)	85 (48.0%)
High (27–40)	6 (2.6%)	4 (66.7%)	2 (33.3%)

GPA, grade point average; PHQ-9, the Patient Health Questionnaire 9; GAD-7, General Anxiety Disorder 7; PSS-10, the Perceived Stress Scale 10. ^a^ Major ward rotation: internal medicine, surgery, obstetrics and gynecology, and pediatrics. ^b^ Minor rotation: ophthalmology; ear, nose, and throat (ENT); family medicine; psychiatry; orthopedics; and rehabilitation.

**Table 2:** Factors associated with the impostor phenomenon among medical clinical students at Chiang Mai University.

Variables	Univariable Analysis		Multivariable Analysis	
	OR (95% CI)	*p*-Value	aOR (95% CI)	*p*-Value
Gender				
Male (ref)	1			
Female	1.043 (0.614–1.77)	0.876		
Year of training				
4th year (ref)	1			
5th year	1.330 (0.714–2.48)	0.369		
6th year	1.482 (0.604–3.64)	0.390		
Ward rotations				
Major	1.250 (0.735–2.12)	0.410		
Minor (ref)	1			
Birth order				
First-born child	1.139 (0.666–1.95)	0.635		
Non-first-born child (ref)	1			
Participants with siblings				
Yes	0.916 (0.495–1.69)	0.779		
No (ref)	1			
Place of training				
Medical school (ref)	1			
Affiliated medical school 1	0.461 (0.179–1.19)	0.109		
Affiliated medical school 2	0.714 (0.330–1.55)	0.393		
GPA				
2.00–2.49	0.653 (0.176–2.42)	0.524		
2.50–2.99	1.771 (0.860–3.65)	0.121		
3.00–3.49	0.819 (0.443–1.52)	0.526		
3.50–4.00 (ref)	1			
Depression				
No	1			
Yes	7.667 (2.833–20.751)	<0.001 *	4.219 (1.448–12.290)	0.008 *
Anxiety				
No	1		1	
Yes	14.241 (3.254–62.323)	<0.001 *	6.462 (1.374–30.392)	0.018 *
Stress				
No	1		1	
Yes	3.034 (1.475–6.244)	0.003 *	2.315 (1.105–4.853)	0.026 *

OR, odds ratio; aOR, adjusted odds ratio; CI, confidence interval; GPA, grade point average. * *p*-value < 0.05.

## Data Availability

De-identified raw data supporting the conclusions of this article will be made available by the authors on reasonable request.

## Supplementary Materials

The following supporting information can be downloaded at: https://www.mdpi.com/article/10.3390/bs13120976/s1, Table S1: Reliability and validity of the Clance Impostor Phenomenon Scale (CIPS) Thai version.
