# Supplementary material for: Exploring Factors Affecting Impostor Syndrome among Undergraduate Clinical Medical Students at Chiang Mai University, Thailand: A Cross-Sectional Study
Source: Behav Sci (Basel). 2023 Nov 27;13(12):976. doi: 10.3390/bs13120976 (PMC10740738; doi:10.3390/bs13120976)
Supplement: Supplementary file 1 [file behavsci-13-00976-s001.zip › behavsci-2712457-supplementary.pdf]

Table S1. Reliability and validity of the Clance Impostor Phenomenon Scale (CIPS) Thai version.

Item	Cronbach's alpha coefficients (N=228)			Test-retest reliability (N=30)	
	Item-test correlation	Item-rest correlation	alpha	ICC	*p*-value
1	0.3099	0.2562	0.9169	0.4957	0.002
2	0.1224	0.0553	0.9210	0.7864	<0.001
3	0.6617	0.6103	0.9102	0.6954	<0.001
4	0.7383	0.6981	0.9081	0.4807	0.001
5	0.5846	0.5304	0.9120	0.6090	<0.001
6	0.7697	0.7284	0.9070	0.8146	<0.001
7	0.6345	0.5786	0.9109	0.5203	1.000
8	0.4635	0.4076	0.9145	0.4082	0.012
9	0.3898	0.3233	0.9164	0.4805	0.003
10	0.6079	0.5543	0.9115	0.6321	<0.001
11	0.5899	0.5349	0.9119	0.5906	<0.001
12	0.6799	0.6304	0.9097	0.7938	<0.001
13	0.7910	0.7552	0.9065	0.8701	<0.001
14	0.7789	0.7452	0.9071	0.5785	<0.001
15	0.8046	0.7705	0.9060	0.5907	<0.001
16	0.5402	0.4807	0.9132	0.6077	<0.001
17	0.6773	0.6235	0.9098	0.8121	<0.001
18	0.7752	0.7406	0.9072	0.7102	<0.001
19	0.6081	0.5455	0.9119	0.4251	0.009
20	0.6563	0.6029	0.9103	0.7939	<0.001
**Scale alpha coefficient**			0.9153		
**CFI**			0.096		
**TLI**			0.829		
**RMSEA**			0.809		

ICC, intraclass correlation; CFI, Comparative Fit Index; TLI, Tucker-Lewis Index; RMSEA, Root Mean Square Error of Approximation
